# The retinol-binding protein receptor STRA6 and melanin cooperatively sustain retinoid signaling and outer blood-retinal barrier integrity

**DOI:** 10.1016/j.jbc.2025.110846

**Published:** 2025-10-22

**Authors:** Aicha Saadane, Johannes von Lintig

**Affiliations:** Department of Pharmacology, School of Medicine, Case Western Reserve University, Cleveland, Ohio, USA

**Keywords:** metabolism, retinoids, lipid transport, epithelia, barrier function

## Abstract

Disruption of the outer blood-retinal barrier (oBRB) is a central feature of retinal degenerative diseases, including age-related macular degeneration, yet the molecular mechanisms maintaining this barrier in the adult eye remain poorly defined. STRA6, a high-affinity receptor for retinol-binding protein (RBP4), mediates vitamin A uptake at the basolateral membrane of the retinal pigment epithelium (RPE), while melanin protects ocular retinoid stores from photooxidative stress. We previously showed that STRA6 deficiency leads to downregulation of junctional proteins in the RPE. Here, we demonstrate that STRA6 and melanin act synergistically to preserve the integrity of the oBRB. In albino Stra6 knockout mice, ocular retinoid levels were severely reduced despite normal circulating retinol levels, and dietary vitamin A delivered *via* chylomicrons failed to compensate for the loss of RBP4-mediated transport. This led to a functional impairment of both rod- and cone-mediated responses, even under vitamin A–sufficient conditions. Mice also showed downregulated tight junction proteins (ZO-1, Claudin-1, Claudin-3), RPE disorganization, barrier leakage, and immune cell infiltration into the subretinal space. These defects were further exacerbated under dietary vitamin A restriction. Importantly, systemic treatment with the pan-retinoic acid receptor (RAR) agonist TTNPB restored junctional gene expression and oBRB function in *Stra6*^*−/−*^ mice, providing evidence that barrier failure arises from impaired retinoid signaling rather than structural loss of STRA6 and melanin. These findings define a novel role for retinoic acid in sustaining RPE barrier function and highlight the combined importance of STRA6-mediated transport and melanin-dependent photoprotection in retinal homeostasis.

The retinal pigment epithelium (RPE) is a monolayer of highly specialized, polarized epithelial cells that perform essential functions for maintaining retinal homeostasis and photoreceptor viability. Situated at the interface of the choroidal vasculature and the neural retina, the RPE mediates directional transport of nutrients, metabolites, and waste products (1, 2, 3, 4). Beyond nutrient handling, the RPE facilitates daily phagocytosis of photoreceptor outer segment discs (5, 6), regulates iron and lipid homeostasis (7), and secretes trophic factors, including vascular endothelial growth factor (VEGF) and pigment epithelium–derived factor (PEDF), to support both the choriocapillaris and photoreceptors (8).

In addition to its metabolic and trophic functions, the RPE establishes the outer blood-retinal barrier (oBRB), a critical structure that preserves the immune-privileged environment of the subretinal space (9). Apical tight junction complexes composed of zonula occludens-1 (ZO-1), claudins, and occludins limit paracellular permeability and prevent infiltration of plasma proteins and immune cells (10). Polarized distribution of transporters and receptors supports a selective transcellular exchange across the epithelium (11). Compromise of this barrier leads to fluid leakage, subretinal inflammation, and photoreceptor degeneration, key features of retinal diseases such as the age-related macular degeneration (AMD) and diabetic retinopathy (12, 13). The etiology of these blinding diseases is influenced by both genetic and environmental factors (14, 15). However, the molecular pathways that govern RPE barrier function and maintenance remain incompletely understood.

Recent work from our laboratory has highlighted a putative role of the vitamin A transporter STRA6 in RPE barrier function (16). STRA6 is an integral membrane protein that facilitates all-*trans*-retinol uptake from circulating serum retinol binding protein (RBP4) at the basolateral site of the RPE (17, 18). *Stra6*^*−/−*^ mice display variable photoreceptor pathologies that are mitigated during adolescence when dietary vitamin A is available in postprandial chylomicrons (19, 20, 21). Under dietary vitamin A restriction, *Stra6*^*−/−*^ mice display downregulated expression of genes encoding tight junction and adhesion molecules and increased barrier permeability (16), suggesting that STRA6 is a critical component for oBRB maintenance.

We further observed that the absence of melanin pigmentation amplifies the deleterious effects of STRA6 deficiency on ocular retinoid metabolism. Albino *Stra6*^*−/−*^ mice exhibit accelerated retinoid turnover and a light-dependent loss of ocular retinoids when compared to their pigmented counterparts (22). Melanin acts as a critical photoprotective and antioxidant reservoir, and its loss predisposes the RPE to photooxidative stress and inflammation. These observations point to a synergistic interaction between STRA6 and melanin pigmentation in maintaining RPE function and homeostasis.

In this study, we investigate the roles of STRA6-mediated retinoid transport and melanin pigmentation in sustaining RPE barrier integrity. Using genetically engineered mouse models and controlled dietary paradigms, we explore how deficiencies in vitamin A uptake and photoprotective melanin pigmentation converge to promote oBRB breakdown and retinal degeneration. Our study provides mechanistic insights into how retinoid signaling and melanin protect the retina and identify potential novel targets for therapeutic intervention in barrier-related retinopathies.

## Results

### Ocular vitamin A homeostasis is severely impaired in Albino *Stra6*^*−/−*^ mice

**Figure 1:**
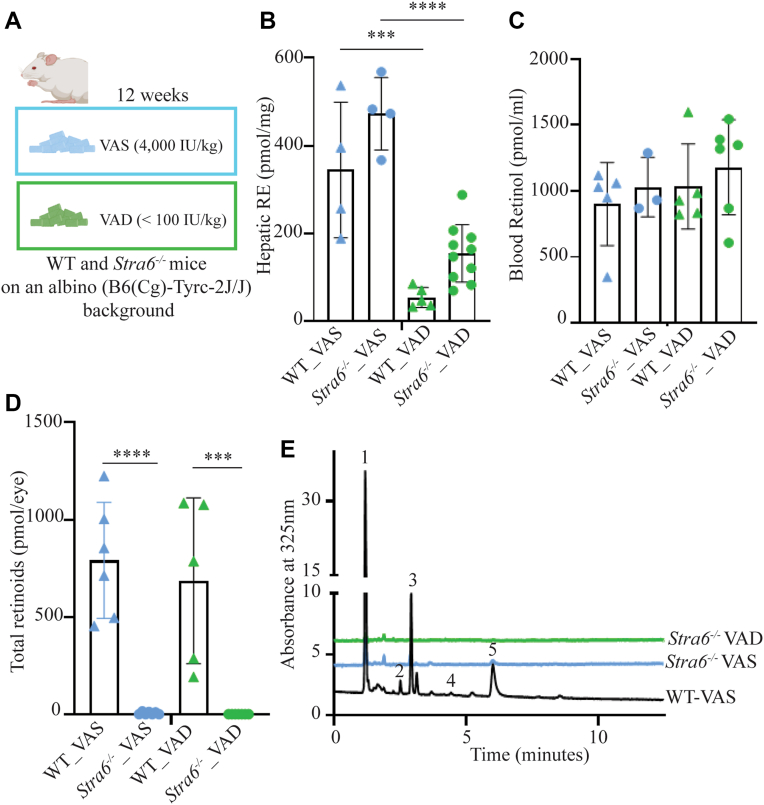
**Retinoid concentrations in selected tissues.** Control and *Stra6*^*−/−*^ mice were fed either a vitamin A-sufficient diet (VAS; 4000 IU/kg) or vitamin A-deficient diet (VAD; < 100 IU/kg) for 12 weeks (*A*). HPLC was used to measure retinoid content in the liver (*B*), serum (*C*), and eyes (*D* and *E*). RE, retinyl ester. Representative HPLC chromatograms illustrating the detection of retinoids in the eyes of control (WT) and *Stra6*^*−/−*^ mice (*E*). peak 1, retinyl esters; peak 2, 11-*cis*-retinal-oxime (syn); peak 3, all-*trans*-retinal-oxime (syn); peak 4, 11-*cis*-retinal-oxime (anti); and peak 5, all-*trans*-retinal-oxime (anti). Data are presented as mean ± SD; n = 4 to 10 mice. Statistical significance was assessed using one-way ANOVA: ∗∗∗*p* < 0.001, ∗∗∗∗*p* < 0.0001. The individual data points in bar graphs represent biological replicates. *Blue* triangles present WT and *blue* dots present *Stra6*^*−/−*^ mice on VAS diet. *Green* triangles present WT and *green* dots present Stra6^−/−^ mice on VAD diet.

Albino *Stra6*^*−/−*^ mice (22) and control albino mice (B6 (Cg)-Tyr^c-2J^/J) were raised on standard chow. At 4 weeks of age, mice were switched to a purified rodent diet containing either 4000 IU/kg vitamin A (VAS) or <100 IU/kg vitamin A (VAD) (Fig. 1*A*). Both groups of mice displayed normal weight gain and fur quality throughout the dietary intervention. After 12 weeks, we quantified retinoid levels by HPLC to assess vitamin A status of mice. Hepatic retinyl ester (RE) stores were abundant in VAS-fed mice of both genotypes, confirming sufficient dietary vitamin A intake. Under VAD conditions, hepatic RE levels were markedly reduced in both genotypes, reflecting mobilization of liver stores to maintain systemic vitamin A homeostasis (Fig. 1*B*). Circulating all-*trans*-retinol concentrations remained comparable between genotypes on either diet, indicating that plasma vitamin A homeostasis was preserved through hepatic release of retinol under both dietary conditions (Fig. 1*C*).

In the eye, albino control mice displayed comparable retinoid levels under both dietary conditions, consistent with efficient vitamin A uptake *via* STRA6-mediated uptake of retinol from serum holo-RBP4 into the RPE. In contrast, albino *Stra6*^*−/−*^ mice lacked ocular retinoids under both VAS and VAD conditions (Fig. 1, *D* and *E*). These findings indicated that STRA6 is essential to sustain ocular vitamin A homeostasis in albino mice, even under VAS diet, underscoring the inability of chylomicron-derived vitamin A to support ocular retinoid homeostasis. This contrasts studies with pigmented *Stra6*^*−/−*^ mice, where melanin-mediated protection of ocular retinoids allows partial compensation through chylomicron-mediated vitamin A delivery to the eyes (16, 23), highlighting a combined role of STRA6 and pigmentation in safeguarding ocular vitamin A levels.

### Scotopic and photopic visual function are severely impaired in Albino *Stra6*^*−/−*^ mice

**Figure 2:**
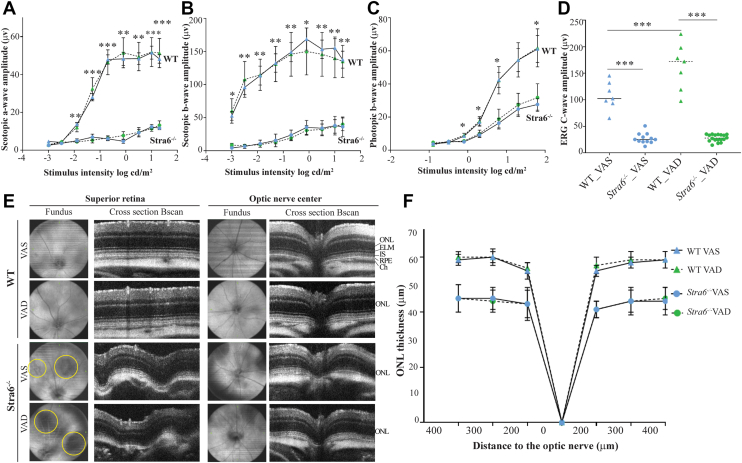
**Retinal function and gross morphology of *Stra6*^*−/−*^ and control mice raised on different diets.** Electroretinography (ERG) was used to assess retinal function, including scotopic (*A* and *B*), photopic (*C*), and c-wave responses (*D*). Retinal morphology was assessed by spectral-domain optical coherence tomography (SD-OCT) (*E*), and outer nuclear layer (ONL) thickness measurements (*F*). Data were collected from 8 to 12 mice per genotype and diet. *Yellow* circles highlight choroidal coloboma. ELM, external limiting membrane; IS, inner segment; RPE, retinal pigment epithelium; Ch, choroid. Values presented as mean ± SEM. Statistical significance was determined using a 2-way repeated measures ANOVA for ERG and a 2-way ANOVA for ONL thickness: ∗*p* < 0.05, ∗∗*p* < 0.01, ∗∗∗*p* < 0.001. *Blue* triangles present WT and *blue* dots represent *Stra6*^*−/−*^ mice on VAS diet. *Green* triangles present WT and *green* dots present *Stra6*^*−/−*^ mice on VAD diet. The individual data points in *panel D* represent biological replicates.

To assess the functional impact of disrupted ocular vitamin A homeostasis, we performed electroretinography (ERG) under dark-adapted (scotopic) and light-adapted (photopic) conditions. In control mice, scotopic a- and b-wave amplitudes were robust and unaffected by dietary vitamin A levels, indicating that both rod photoreceptor function and downstream retinal circuitry remained intact under VAS and VAD conditions. In stark contrast, *Stra6*^*−/−*^ mice exhibited markedly reduced scotopic a- and b-wave amplitudes under both diets (Fig. 2, *A* and *B*), demonstrating that chylomicron-derived retinoids under VAS diet could not sustain photoreceptor responsiveness in the absence of STRA6 as previously reported for pigmented *Stra6*^*−/−*^ mice (16).

Photopic ERG recordings revealed preserved cone-mediated b-wave amplitudes in control albino mice regardless of diet, whereas *Stra6*^*−/−*^ mice lacked photopic responses under both VAS and VAD conditions (Fig. 2*C*). A lack of photopic ERG response has been previously reported in pigmented STRA6-deficient mice and is consistent with the critical role of STRA6 in providing chromophore supplies to cone photoreceptors, even when dietary vitamin A is abundant (20, 24).

We also recorded ERG c-waves, which originates from RPE and reflects the resulting hyperpolarization of the RPE cells' apical membrane (25). *Stra6*^*−/−*^ mice exhibited severely diminished c-wave amplitudes compared to controls under both diets (Fig. 2*D*). Given that c-wave generation depends on photoreceptor light absorption and subsequent extracellular potassium shifts, this deficit underscores that STRA6-mediated vitamin A transport is critical not only for phototransduction but also for maintaining RPE physiology in the adult eye.

### Photoreceptor degeneration in *Stra6*^*−/−*^ mice

To assess the impact of diet and genotype on retinal morphology, we performed noninvasive imaging using SD-OCT and SLO. A subset of *Stra6*^*−/−*^ mice exhibited choroidal coloboma, irrespective of dietary vitamin A levels during the experimental period (Fig. 2*E*). These lesions were predominantly localized to the superior (dorsal) retina and have been previously reported in pigmented *Stra6*^*−/−*^ mice at postnatal day 21 (19), consistent with a developmental origin. Notably, such abnormalities were never observed in control albino mice, indicating that the lack of melanin alone does not contribute to this phenotype.

Cross-sectional SD-OCT imaging revealed additional structural defects in albino *Stra6*^*−/−*^ mice beyond the coloboma regions. The external limiting membrane (ELM) was absent, and the distinction between outer retinal layers, including the inner and outer segments, was lost (Fig. 2*E*). Within areas affected by coloboma, these abnormalities were more severe, with poorly defined RPE and choroidal layers (Fig. 2*E*). Quantitative analysis confirmed significant photoreceptor degeneration, evidenced by a marked reduction in outer nuclear layer (ONL) thickness in albino *Stra6*^*−/−*^ mice as compared to controls (Fig. 2*F*). This reduction of the ONL thickness was observed under both dietary conditions and was previously not observed in pigmented *Stra6*^*−/−*^ mice (16), underscoring that they resulted from a combination of the lack of melanin and STRA6. Together, these findings highlight the critical role of STRA6 in maintaining photoreceptor integrity and survival in albino mice even when vitamin A is abundant in the diet.

### Immune cells activation in STRA6-Deficient mice

**Figure 3:**
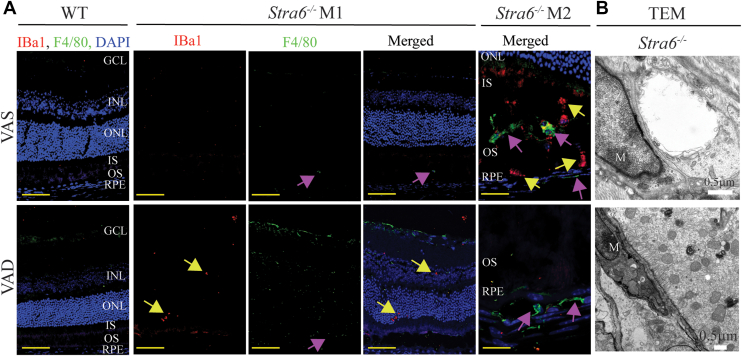
**Photoreceptor cell degeneration and immune cell activation in STRA6-deficient mice.***A*, representative immunostaining of cross-retinal sections for Iba1 (*red*, *yellow arrows*) and F4/80 (*green*, *fuchsia arrows*) in control (WT) and *Stra6*^*−/−*^ mice maintained on vitamin A-sufficient (VAS) or vitamin A-deficient (VAD) diets. Nuclei were counterstained with DAPI. Scale bar = 50 μm. Sample size: WT (*n* = 2), *Stra6*^*−/−*^ (*n* = 4). *B* Representative transmission electron microscopy (TEM) images showing macrophages (M) near Bruch’s membrane. Scale bar = 0.5 μm. GCL, ganglion cell layer; INL, inner nuclear layer; ONL, outer nuclear layer; IS, inner segment; OS, outer segment; RPE, retinal pigment epithelium, TEM, transmission electron microscopy; M, macrophages; M1 and M2 are representative immunostainings from two different *Stra6*^*−/−*^ mice.

Photoreceptor cell degeneration, as observed here in albino *Stra6*^*−/−*^ mice, is often accompanied by significant activation of resident immune cells (microglia) and infiltration of macrophages (26, 27, 28). To investigate whether this pathology occurred in STRA6-deficient albino mice, we stained retinal cross-sections with Iba1, a microglia marker, and F4/80, a macrophage marker. In albino *Stra6*^*−/−*^ mice, we observed a marked activation and migration of microglia, along with substantial macrophage infiltration into the outer retina, a normally immune-privileged region (29, 30) (Fig. 3*A*). TEM confirmed the presence of macrophages in the vicinity of Bruch’s membrane under both dietary conditions (Fig. 3*B*).

**Figure 4:**
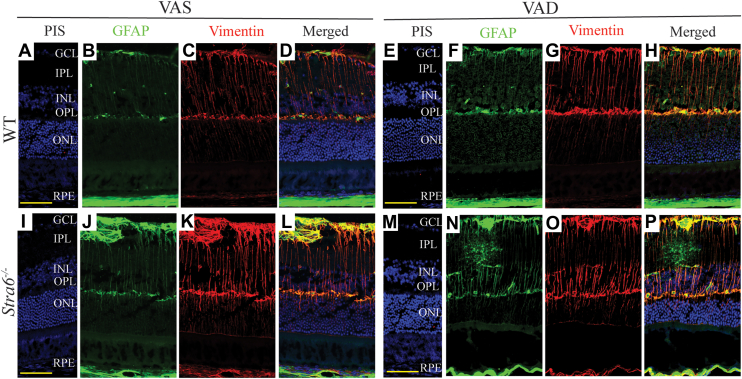
**Glial cell activation in *Stra6*^*−/−*^ mice.** Representative immunofluorescence images of glial fibrillary acidic protein (GFAP) and vimentin in *Stra6*^*−/−*^ and control (WT) mice maintained on either a vitamin A-sufficient (VAS) or vitamin A-deficient (VAD) diet. Immunostaining with GFAP (*green*; *B*, *F*, *J*, and *N*) and vimentin (*red*; *C*, *G*, *K*, and *N*) highlights reactive changes in glial cells. *PanelsA*, *E*, *I*, and *M* indicate control stains using nonimmune serum (PIS). Scale bar = 50 μm. GCL, ganglion cell layer; INL, inner nuclear layer; ONL, outer nuclear layer; IS, inner segment; OS, outer segment; RPE, retinal pigment epithelium.

Because photoreceptor degeneration is often accompanied by glial cell activation and neuroinflammation (31), we examined retinal cross-sections from control and *Stra6*^*−/−*^ mice using vimentin and GFAP immunostaining to assess Müller cell gliosis and astrocytic activation. In control mice, vimentin labeling revealed normal Müller cell processes spanning from the nerve fiber layer (NFL) to the inner nuclear layer (INL), and GFAP staining was largely absent under vitamin A sufficient (VAS) conditions. Only a minimal increase in GFAP expression was observed in the ganglion cell layer (GCL) and NFL of VAD-fed controls (Fig. 4, *B*–*D*, *F*–*H*), indicating that dietary vitamin A restriction alone was insufficient to induce significant glial activation.

In sharp contrast, retinas of *Stra6*^*−/−*^ mice displayed pronounced upregulation of both vimentin and GFAP, particularly within the GCL, NFL, and inner plexiform layer (IPL) (Fig. 4, *J*–*L*, *N*–*P*). Under VAD conditions, these changes were markedly exacerbated. Müller cell processes appeared fragmented and disorganized, with vimentin labeling reduced in the IPL and abnormally extended into the outer nuclear layer (ONL) (Fig. 4*O*). GFAP expression was further elevated in albino *Stra6*^*−/−*^ mice on the VAD diet, extending beyond Müller cells and prominently localizing around blood vessels (Fig. 4, *N* and *P*), consistent with activation of astrocytes and perivascular glia. Importantly, these pathological changes were not observed in control mice under either dietary condition.

Together, these findings demonstrate that STRA6 deficiency triggers robust glial activation and neuroinflammation in the retina, and that vitamin A restriction amplifies these processes, indicating that under VAS conditions dietary retinoids in chylomicrons mitigates the pathology and improves retinal immune homeostasis.

### Breakdown of the oBRB in Albino *Stra6*^*−/−*^ mice fed a VAD diet

**Figure 5:**
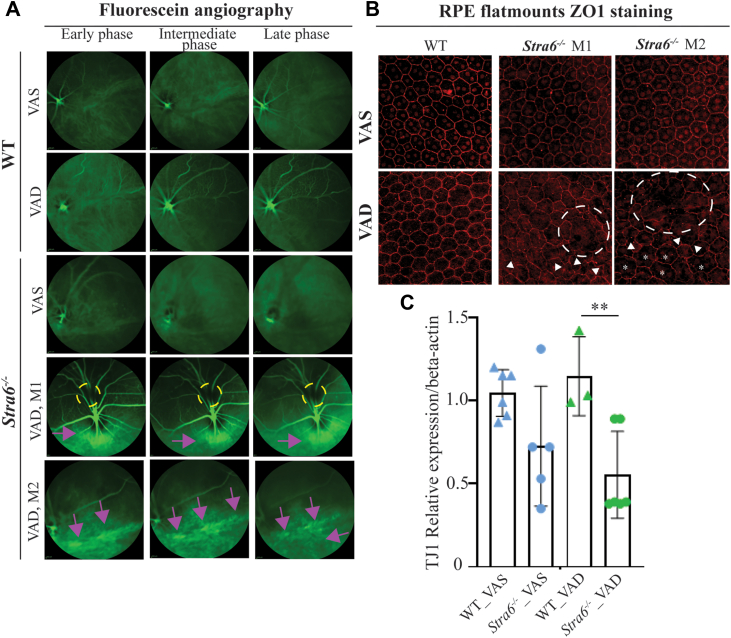
**Breakdown of the outer blood-retinalbarrier in *Stra6*^*−/−*^ mice.***A*, Representative images of fluorescein angiography (FA) in *Stra6*^*−/−*^ and control (WT) mice fed either a vitamin A-sufficient (VAS) or a vitamin A-deficient (VAD) diet (*n* = 8–13). *Yellow* circles highlight areas of non-perfusion, while *fuchsia arrows* denote regions where fluorescent dye breaches beneath the retinal pigment epithelium. *B* Flat mounts of the retina pigment epithelium stained with anti-Zo-1 antibodies (*red*). *C*, qRT-PCR analysis of mRNA levels of Tight Junction 1 (TJ1) in control (WT) and *Stra6*^*−/−*^ mice. Beta-actin was used as a housekeeping gene. Values are presented as means ± SD. Statistical significance was determined using one-way ANOVA. ∗∗*p* < 0.001M1 and M2 are representative immunostainings from two different mice. The individual data points in *panel C* represent biological replicates.

We previously demonstrated that genes critical for epithelial barrier maintenance are downregulated in pigmented *Stra6*^*−/−*^ mice under VAD conditions (16). The lack of melanin pigmentation in albino mice allows live imaging of fluorescein leakage in the outer retina. Therefore, to determine whether the neuroinflammatory phenotype in albino *Stra6*^*−/−*^ mice arises from oBRB breakdown, we employed *in vivo* fluorescein angiography (FA). Using scanning laser ophthalmoscopy (SLO), FA images were acquired during early, intermediate, and late phases after fluorescein dye injection. In *Stra6*^*−/−*^ mice on a VAD diet, angiograms revealed prominent areas of hyperfluorescence that intensified over time (Fig. 5*A*). These hyperfluorescent regions indicate fluorescein leakage across a compromised RPE into the outer retina, reflecting a breach of the normal barrier to fluorescein imposed by the RPE junctions. Leakage presented either as large focal zones of hyperfluorescence (Fig. 5*A*, mouse 1; M1) or as clusters of smaller hyperfluorescent spots extending over broader retinal regions (Fig. 5*A*, mouse 2; M2). These findings directly correlate with the increased immune cell infiltration and glial activation observed in *Stra6*^*−/−*^ mice on VAD, consistent with barrier breakdown triggering local inflammation.

In contrast, no fluorescein leakage was observed in *Stra6*^*−/−*^ mice on a VAS diet or in control mice under either dietary condition (Fig. 5*A*), indicating that oBRB integrity was preserved in these groups. Areas of non-perfusion were also detected in *Stra6*^*−/−*^ mice irrespective of diet, likely reflecting colobomas characteristic of this genotype (Fig. 2*E*), but were absent in control mice.

To confirm that the hyperfluorescence observed in fluorescein angiography (FA) reflected disruption of the oBRB, we analyzed the integrity of RPE tight junctions by immunostaining for Zonula Occludens-1 (ZO-1), a key adhesion protein essential for oBRB maintenance (9). In wild-type mice, ZO-1 staining displayed the characteristic hexagonal pattern delineating intact RPE cell borders under both VAS and VAD dietary conditions (Fig. 5*B*), indicating preserved tight junction integrity.

In *Stra6*^*−/−*^ mice on a VAS diet, RPE cells largely retained their normal morphology, but ZO-1 staining appeared irregular and patchy, suggesting partial compromise of tight junctions (Fig. 5*B*). Strikingly, *Stra6*^*−/−*^ mice on a VAD diet exhibited severe tight junction disruption, with large gaps in ZO-1 labeling and misshapen RPE cells. Two distinct patterns of ZO-1 loss were identified: (i) widespread tight junction breakdown affecting multiple contiguous RPE cells (Fig. 5*B*, dotted circles), corresponding to the large hyperfluorescent areas detected in FA (Fig. 5*A*, mouse 1; M1), and (ii) focal disruptions (Fig. 5*B*, white arrow heads and white asterisks) manifesting as isolated or clustered “window defects” in FA (Fig. 5*A*, M2, fuchsia arrows).

**Figure 6:**
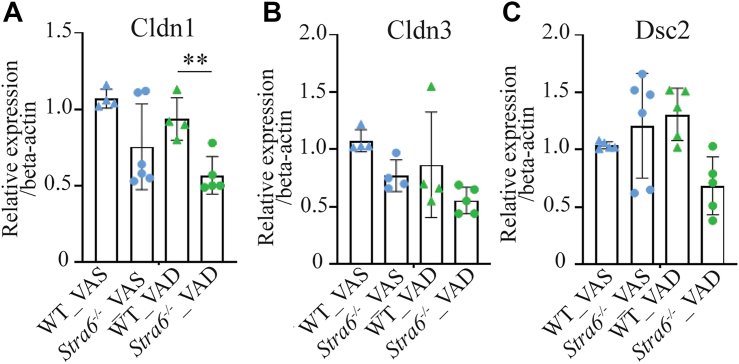
**Tight junction-related genes are downregulated in VAD *Stra6*^*−/−*^ mice.** Deficiencies in STRA6 and melanin, combined with vitamin A restriction, resulted in a significant decrease or trend towards decreasing mRNA levels of genes encoding tight junction proteins: qRT-PCR analysis was conducted with ocular RNA preparation from 4 to 6 mice per genotype and dietary conditions. Beta-actin was used as a housekeeping gene. Claudin 1 (*Cldn1*) (*A*), Claudin 3 (*Cdln3*) (*B*), and Desmocollin 2 (*Dsc2*) (*C*). Values are presented as means ± SD. Statistical significance was determined using one-way ANOVA. ∗∗*p* < 0.001. The individual data points in the bar graphs present biological replicates. *Blue* triangles present WT and *blue* dots represent *Stra6*^*−/−*^ mice on VAS diet. *Green* triangles present WT and *green* dots present *Stra6*^*−/−*^ mice on VAD diet.

These structural changes were further supported by quantitative PCR analysis, which revealed significant downregulation of the Tight Junction Protein 1 (Tjp1) gene encoding ZO-1 in *Stra6*^*−/−*^ mice on a VAD diet (Fig. 5*C*), along with decreased expression of *Claudin-1* and *Claudin-3* (Fig. 6). The observed barrier failure is consistent with and likely contributes to the neuroinflammatory phenotype in *Stra6*^*−/−*^ mice, as loss of RPE tight junctions facilitates immune cell infiltration and glial activation. Together, these findings demonstrate that in the absence of STRA6, dietary vitamin A restriction leads to oBRB failure, characterized by tight junction loss, RPE cell disorganization, and fluorescein leakage.

### Ultrastructural changes in the choriocapillaris, Bruch’s membrane, and RPE of *Stra6*^*−/−*^ mice

**Figure 7:**
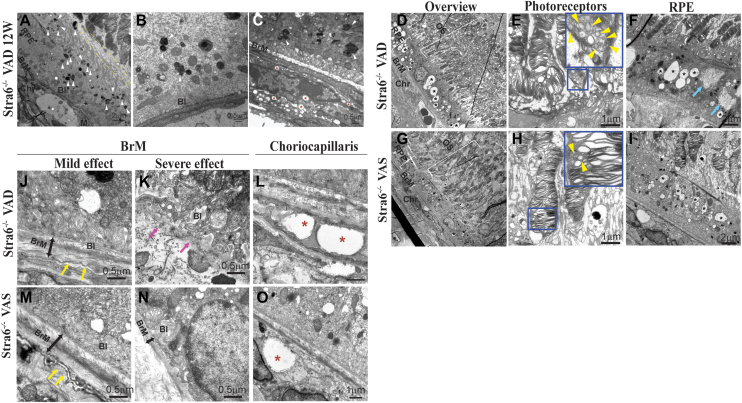
**Ultrastructural changes of the retina of *Stra6*^*−/−*^ mice under different diets.** Transmission electron microscopy (TEM) was used to examine the ultrastructural changes in *Stra6*^*−/−*^ albino mice maintained on a vitamin A-deficient (VAD) diet for 3 months (*A*–*C*) and on either a vitamin A-sufficient (VAS) or VAD diet for 5 months (*D*–*O*). Please note that panel I is an overview of the region displayed in panel *O* and in panel *B* of Figure 3. Inset *blue* boxes in E and (*H*) highlight enlarged views of outer segments in *Stra6*^*−/−*^ mice. Scale bars are as indicated on micrographs, ranging from 0.5 μm to 2 μm. Ch, choroid; BrM, Bruch’s membrane; BI, basal infoldings; REP, retinal pigment epithelium; OS, outer segments.

We next used transmission electron microscopy (TEM) to study the ultrastructure of the outer retina and the choriocapillaris of *Stra6*^*−/−*^ mice. After 12 weeks on VAD diet, *Stra6*^*−/−*^ albino mice showed disorganized OS, and abnormal microvilli which were separated from OS by a wall of membranous debris (Fig. 7*A*, yellow dashed ellipse). The RPE was filled with phagosomes which were incompletely digested (Fig. 7, *A*–*C*, white arrowheads) and Bruch’s membrane (BrM) lost their pentamer laminar appearance and had electro-lucent aspect (Fig. 7*C*). Abnormalities were also seen in the choriocapillaris with macrophages adjacent to BrM, the presence of vacuoles and empty spaces (Fig. 7, *C*, *L* and *O*, red asterisks). These structural abnormalities prompted us to investigate whether prolonged vitamin A restriction over a 5-month period would exacerbate the observed defects when compared to age-matched mice on VAS diet. Lower magnification images showed a disorganized photoreceptors outer segment (OS) with disordered and vacuolated disk membranes in *Stra6*^*−/−*^ albino mice (Fig. 7, *D*, *E*, and *G*–*H*, yellow arrowheads).

The RPE of these mice were filled with vacuoles of various sizes, however the number and the size of these vacuoles were higher under VAD when compared to VAS diet (Fig. 7, *F* and *I*; black asterisks). In addition, membranes-filled structures with irregular borders were also present under VAD diet (Fig. 7*F*; blue arrows). Basal infoldings were mildly affected under VAS diet (Fig. 7, *M* and *N*). However, the basal infoldings were severely disorganized, thickened, and in some instances were completely depleted in *Stra6*^*−/−*^ mice raised on VAD diet (Fig. 7, *J* and *K*). BrM structure was altered in *Stra6*^*−/−*^ mice, regardless of the diet, but under VAD mice was more severely affected, and BrM was thinner and, in some instances, completely absent (Fig. 7, *J* and *K*; double ended arrows and fuchsia arrows). Moreover, the choriocapillaris fenestrations were not clearly delineated under VAD when compared to VAS diet (Fig. 7, *J* and *M*; yellow arrows).

### Treatment with a RAR agonist prevents downregulation of TJ1 and oBRB dysfunction

Our previous (16) and current observations indicate that oBRB integrity is progressively compromised in *Stra6*^*−/−*^ mice, with barrier leakage becoming increasingly pronounced under VAD conditions. This graded phenotype suggests that dietary vitamin A in chylomicrons can maintain oBRB in STRA6-deficiency, but as vitamin A supply gets abolished under VAD, retinal pathology intensifies. These findings implicate a key role for retinoid-dependent mechanisms in maintaining barrier function. Retinoid signaling is known to support epithelial tight junction integrity in zebrafish (32) and has been implicated in the immune-privileged status of the RPE (33). However, the critical question remained whether the oBRB breakdown in *Stra6*^*−/−*^ mice is a direct consequence of a loss of retinoid signaling.

**Figure 8:**
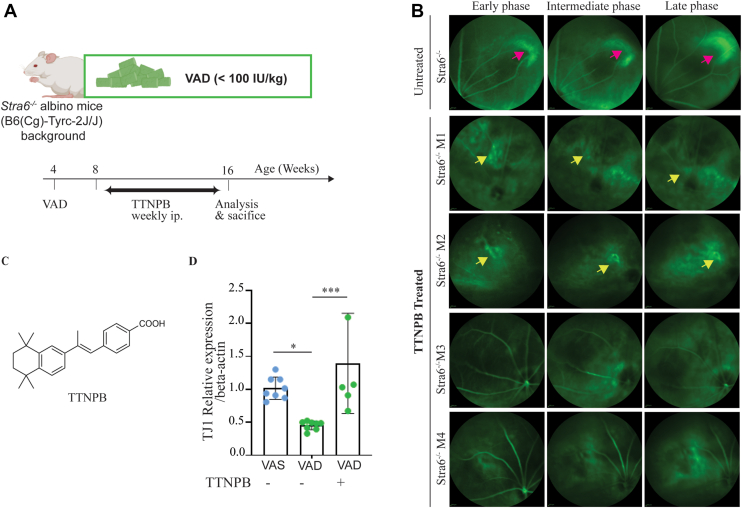
**Retinoic acid agonist treatment restoresouter blood-retinal barrierfunction.***A*, after weaning albino *Stra6*^*−/−*^ mice raised on chow were subjected to VAD diet for 12 weeks, 4 weeks into VAD, mice start receiving weekly RA agonist (TTNPB, C) for a total of 8 weeks. Data were collected from 5 to 8 mice. *B* Representative fundus fluorescein angiography of *Stra6*^*−/−*^ albino mice (n = 5) treated with RA analog at early, intermediate, and late phases. *Yellow arrow*s indicate window defects. *C* indicate the structure of TTNPB. *D*, relative gene expression of Tight junction (TJ1) normalized to beta-actine, the house keeping gene. Values are presented as mean ± SD. Statistical significance was determined using one-way ANOVA. ∗*p* < 0.05, ∗∗*p* < 0.001. The individual data points in *panel D* present biological replicates. *Blue* dots present *Stra6*^*−/−*^ mice on VAS diet *green* dots present *Stra6*^*−/−*^ mice on VAD diet.

To this end, we tested whether pharmacological activation of retinoid signaling could rescue the phenotype. In this experiment, albino *Stra6*^*−/−*^ mice were maintained on a VAD diet for 4 weeks to induce retinoid depletion and oBRB disruption, then treated with the pan-retinoic acid receptor (RAR) agonist TTNPB *via* weekly intraperitoneal injections for 8 weeks (Fig. 8, *A* and *C*). Untreated *Stra6*^*−/−*^ mice on VAD served as controls. At 16 weeks of age, fluorescein angiography revealed that TTNPB treatment markedly reduced barrier leakage; of five treated *Stra6*^*−/−*^ mice, three displayed normal angiograms, while two exhibited mild residual hyperfluorescent defects (Fig. 8*B*). In contrast, all untreated *Stra6*^*−/−*^ mice showed oBRB leakage (Fig. 8*B*). Consistent with restored barrier function, TTNPB administration also prevented downregulation of *Tjp1* expression in *Stra6*^*−/−*^ mice relative to untreated *Stra6*^*−/−*^ mice (Fig. 8*D*).

These findings showed that TTNBP treatment ameliorates oBRB breakdown in *Stra6*^*−/−*^ mice under VAD and suggest retinoic acid as a key regulator of RPE barrier integrity and homeostasis in the adult mouse eye.

## Discussion

This study establishes STRA6-mediated vitamin A transport and melanin-dependent photoprotection as synergistic mechanisms essential for maintaining the integrity of the oBRB and photoreceptor function in the adult eye. Using albino *Stra6*^*−/−*^ mice, we show that dietary vitamin A delivered *via* chylomicrons is insufficient to sustain ocular retinoid homeostasis in the absence of STRA6. This failure results in progressive retinoid depletion, loss of tight junction integrity, photoreceptor degeneration, and neuroinflammation. The phenotype is markedly exacerbated under VAD conditions, underscoring the vulnerability of the oBRB to declining retinoid availability when both STRA6 and melanin are absent. This contrast, our findings in pigmented mice where ocular VAD was less pronounced and retinal degeneration was prevented by retinoids in chylomicrons (16). Importantly, activation of retinoid signaling with the pan-retinoic acid receptor agonist TTNPB improved barrier function and suppressed inflammation in albino *Stra6*^*−/−*^ mice, providing evidence that retinoid signaling is essential for maintaining RPE integrity. These findings not only reveal a previously unappreciated role for RA in adult retinal homeostasis but also suggest that impaired retinoid signaling may contribute to barrier dysfunction in broader disease contexts of the eye.

Melanin acts as a light filter and quencher of reactive oxygen species (14, 34), shielding ocular retinoids from photooxidative stress and limiting light-induced degradation of retinoids and their conjugation products (22, 35). In albino *Stra6*^*−/−*^ mice, the absence of melanin accelerates retinoid turnover and exacerbates the consequences of impaired vitamin A transport (22). Chromophore deficiency in photoreceptors leads to the accumulation of unliganded opsins, which are known to generate oxidative stress (36, 37). This oxidative burden is likely magnified in albino mice, where the absence of melanin removes a key photoprotective mechanism. This concept is supported by findings from Sparrow and colleagues (35, 38, 39), revealing that albino *Abca4*^*−/−*^ mice with impaired ocular retinoid metabolism exhibit more severe pathology than their pigmented counterparts, further underscoring the protective role of melanin in retinal homeostasis.

While most studies of VAD-associated retinal degeneration have focused on chromophore depletion and failure of phototransduction (37, 40), our findings extend this paradigm to retinoid signaling. We previously reported that retinoid depletion in pigmented *Stra6*^*−/−*^ mice is associated with downregulation of tight junction genes and oBRB disruption, implicating RA in RPE barrier maintenance (16). The current study evidence for this hypothesis: activation of RARs by TTNBP maintained barrier function and suppressed neuroinflammation in albino *Stra6*^*−/−*^ mice under VAD conditions. This mechanism is consistent with the expression of RARs in the RPE, where they regulate gene networks involved in epithelial polarity, adhesion, and immune regulation, including claudins (*Cldn3* and *Cldn4*), plakophilin-1 (*Pkp1*), and desmocollins (*Dsc2* and *Dsc3*) (16, 32, 41). These findings expand the classical view of vitamin A biology, defining a novel role for retinoids in maintaining RPE integrity and retinal immune homeostasis in adult eyes.

Our findings also have implications for understanding the pathology of vitamin A deficiency. Classical models have focused on chromophore depletion and the accumulation of unliganded opsins in photoreceptors as the primary drivers of visual dysfunction (36, 37). While this mechanism remains valid, especially in models such as *Lrat*- or *Rpe65*-deficient mice (40, 42, 43), our results extend this paradigm by showing that vitamin A deficiency affects RPE barrier integrity and immune privilege. This additional level of pathology results from a disruption of both retinoid signaling and chromophore metabolism while mutation in genes of chromophore synthesis result in milder phenotypes (44). These insights suggest that timely intervention is critical to prevent progression to an irreversible state of retinal degeneration in clinical vitamin A deficiency. Future studies should explore the temporal sequence of these events and determine whether photoreceptor degeneration and barrier breakdown occur independently or are interdependent.

Breakdown of the oBRB also is a pathological hallmark of several retinal diseases, including diabetic retinopathy, central serous chorioretinopathy, and age-related macular degeneration (AMD). In AMD, early functional deficits include a delayed rod mediated dark adaptation, which precedes overt structural degeneration (45). This loss of rod-mediated vision may reflect localized impairment of vitamin A transport in areas of drusen formation, consistent with a role for retinoid deficiency in driving barrier failure and disease progression.

Our findings also underscore the influence of melanin pigmentation on retinal disease susceptibility. Melanin not only protects against light-induced oxidative stress but also modulates visual cycle byproducts, mitigating their toxic effects (22, 34, 35). The heightened vulnerability of albino *Stra6*^*−/−*^ mice to ocular VAD underscores how absence of melanin potentiates retinoid depletion and worsens barrier failure. Epidemiological studies support this concept, showing that AMD prevalence is significantly lower in Black Americans compared to White populations (46, 47), suggesting that pigmentation confers resilience against oxidative stress and metabolic challenges in the aging retina.

In summary, by linking STRA6-mediated vitamin A transport and melanin-dependent photoprotection to barrier maintenance and immune regulation, our study provides mechanistic insight into how these pathways converge to preserve retinal health. These findings may inform future therapeutic strategies aimed at stabilizing RPE barrier function and suppressing maladaptive inflammation in retinal diseases where retinoid metabolism is compromised.

## Experimental procedure

### Animal husbandry

Albino *Stra6*^*−/−*^ mice on a C57BL/6J background were generated previously in this laboratory by cross breeding C57B/6J *Stra6*^*−/−*^ and albino B6(Cg)-Tyr^c-2J^/J control mice (22). The matching control albino mice were purchased from Jackson Laboratories. Mice were bred and maintained on a standard 12-h light (∼10 lux)-dark cycle and water ad libitum. To determine the effects of dietary vitamin A supply on the *Stra6* phenotype, we fed mice three different diets: a purified diet with an AIN-93G formulation containing vitamin A 4000 IU/kg, referred to as vitamin A–sufficient diet (VAS), the same diet without vitamin A supplement (<100 IU/kg), referred to as vitamin A deficient diet (VAD), and rodent breeder chow with vitamin A excess (15,000 IU/kg), referred to as chow diet for normal maintenance of mice. All animal procedures were approved by the Case Western Reserve University Institutional Animal Care and Use Committee and conformed to recommendations of the American Veterinary Association Panel on Euthanasia and the Association for Research in Vision and Ophthalmology. Our study examined male and female animals, and similar findings are reported for both sexes.

The retinoic acid receptor pan-agonist TTNPB, (T3757, Sigma) was dissolved in DMSO (1 mg/ml) and the stock solution was further diluted in PBS and a dose of 0.05 mg/kg was injected intraperitoneally. After weaning, albino *Stra6*^*−/−*^ mice raised on chow were subjected to VAD diet for 12 weeks, 4 weeks into VAD, mice start receiving weekly RA agonist for a total of 8 weeks. The TTNBP dose was chosen based on findings of previous mouse experiments (48). At the end of the dietary intervention, mice were anesthetized by using a drug cocktail of ketamine and xylazine (20 mg/ml, 1.75 mg/ml respectively). Blood was collected *via* cardiac puncture. Mice were transcardially perfused with 20 ml of ice-cold PBS and sacrificed by cervical dislocation and tissues were immediately harvest and stored in −80 °C until analysis.

### Retinal imaging and visual function

We conducted an anatomic characterization of *Stra6*^*−/−*^ and wild type albino mice using spectral domain-optical coherence tomography (OCT; the 840 HHP spectral domain-OCT system; Bioptigen), Laser scanning ophthalmoscope (SLO, Spectralis HRA + OCT; Heidelberg Engineering), and ERG (Diagnosys Celeris rodent ERG device; Diagnosys) recordings as previously described (49). Briefly, for ERG, mice were dark adapted overnight and then anesthetized. The pupils were dilated with 1% tropicamide eye drops. ERGs were recorded using a contact lens electrode placed on each cornea. For scotopic ERG responses, mice were exposed to 10 steps of a white light flash stimulus ranging from 0.001 to 20 cd s m^−2^, and the inter stimulus intervals ranging from 4 s for low luminance flashes to 90 s for the highest stimulus. Scotopic ERG recordings was followed by photopic ERG responses, after 7 min of light adaptation, ERG responses were recorded with strobe-flash (0.32–63 cd s.m^−2^) superimposed on the adapting field. Amplitude values were averaged between right and left eyes.

### Fluorescein angiography

Fluorescein angiography (FA) was as previously described (50) after a bolus (0.1 ml) i.p. injection of 1% sodium fluorescein in phosphate-buffered saline (PBS). Briefly, mice were anesthetized, and eyes were dilated as described above. Sodium fluorescein in PBS was injected intraperitoneally. Retina monitoring started immediately after the injection and continued for up to 15 min. The beam of SLO was focused on the deeper retina and images were acquired at early, intermediate, and late phases.

### HPLC analysis

Retinoid analysis was performed a described (16). Briefly, 10 mg liver was homogenized in 200 μl PBS and one whole eye was homogenized in 200 μl hydroxylamine (2M, pH 6.8), and 100 μl serum was added to 100 μl PBS. Retinoids were extracted using 200 μl methanol, 400 μl acetone and 500 μl hexane. The hexane extraction was once repeated. A normal phase Zorbax Sil (5 μm. 4.6 × 150 mm) column was used for HPLC analysis. Chromatographic separation was achieved by isocratic flow of 10% ethyl acetate/90% hexanes. The HPLC was previously scaled with standard compounds to quantify the molar amounts of retinoids.

### RPE flat mounts and immunostaining

The preparation of choroid/RPE flat mounts was as previously described (16). Briefly, mice were sacrificed, and their eyes were enucleated, followed by fixation in 4% paraformaldehyde (PFA) for 30 min at room temperature. The cornea, lens, and vitreous were removed, and four incisions from outside of the eyecup towards the center were made to flatten the remaining eyecup. Next, the choroid/RPE was separated from neural retina and placed into 4% PFA for an additional 30 min. Choroid/RPE flat mounts were permeabilized and blocked for 24 h at 4 °C with PBS containing 0.5% Triton X-100, 2% bovine serum albumin, and 5% normal goat serum. Subsequent incubation with primary rabbit antibody against ZO1 (442200; Invitrogen, Waltham, MA; 1:100 dilutions in PBS+0.05% Tween-20) was overnight at 4 °C, followed by a 6-h incubation at 4 °C with Hoechst (33342; Invitrogen; 1:1000 dilutions in PBS). RPE flat mounts were then washed for 1 h at 4 °C with four changes of PBS, followed by an overnight incubation at 4 °C in PBS. The next morning, goat anti-rabbit Alexa 594 IgG (111-585-045; Jakson Laboratories, West Grove, PA; 1:200 dilutions) was added. RPE flat mounts were washed for 1 h at 4 °C with four changes of PBS and then mounted with ProLong Gold without DAPI. Images were acquired using the Olympus Fluoview FV1200 Laser Scanning Confocal Microscope (Olympus).

### Eyecup isolation, RNA extraction, and q-PCR

Eyecups were isolated as previously described (16) with few adjustments to accommodate the albino eyes. Briefly, eyes were enucleated, and anterior chamber and vitreous were removed. The eyecups were incubated with 2% dispase for 5 min in 37 °C. The neural retina was peeled off, and the remaining Choroid/RPE complex was rinsed in PBS. The choroid/RPE from two mice were pooled together for RNA isolation. Total RNA (1 μg) from pooled samples of mouse choroid-retina complex was isolated as described using the Trizol Reagent (Thermo Fisher Scientific). This RNA was then transcribed to cDNA by SuperScript III reverse transcriptase (Invitrogen), according to manufacturer's instructions. PCRs were performed in duplicates and were normalized to beta-actin. Mouse primers used for analysis were Actinβ (Mm02619580_g1), *Cldn1* (Mm01342184_m1), *Cldn3* (Mm00515499_s1), *Dsc2* (Mm00516355_m1), *Tj1* (Mm01320638_m1). Changes in relative mRNA level were calculated by the 2−ΔΔCt method.

### Immunohistochemistry

The preparation of frozen retinal sections was as described (50). Retinal sections were warmed to room temperature for 30 min and washed three times for 5 min with PBS containing 0.05% Tween 20. Sections were then permeabilized for 20 min with PBS containing 0.3% Triton X-100 and blocked for 1 h with 5% normal goat serum (Invitrogen) in PBS containing 0.05% Tween 20 (blocking buffer). Sections were incubated overnight at 4 °C with rabbit anti-IBA1 (MA5-36257; Invitrogen; 1:200 dilutions in blocking buffer), F/480 (MCA497GA; Bio-Rad, Radnor, PA; 1:200 in blocking buffer), rabbit anti-GFAP (3670P; 1:400), and anti-vimentin (ab92547; abcam, Waltham, MA; 1:200). The next morning, slides were washed three times for 5 min with PBS containing 0.05% Tween 20 and incubated for 1 h in the dark with the appropriate secondary antibodies: anti-rabbit Alexa Fluor 595, anti-rat Alexa Fluor 647–conjugated, and anti-mouse Alexa Fluor 647 (111-585-045, 111-605-144, and 115-605-003, respectively; Jackson ImmunoResearch, West Grove, PA; 1:200 dilutions in blocking buffer). Sections were washed three times for 5 min with PBS, then dipped three times in distilled water, and next covered with ProLong Gold antifade reagent with DAPI (P36935; Molecular Probes) and protected with a glass coverslip. Images were acquired using the Olympus Fluoview FV1200 Laser Scanning Confocal Microscope (Olympus).

### Statistical analysis

Data are expressed as mean ± SD, except for retinal thickness and ERG measurement that are expressed as mean ± SEM. Statistical analyses were performed with either one-way or two-way ANOVA, except for ERG data, which were analyzed by two-way repeated measures of variance. The results were considered significant at ∗ *p* ≤ 0.05; ∗∗*p* ≤ 0.01; ∗∗∗*p* ≤ 0.001; and ∗∗∗∗*p* ≤ 0.0001.

## Data availability

All data are contained within the article.

## Conflict of interest

The authors declare that they have no conflicts of interest with the content of this article.
